# Blood pressure and lipid management fall far short in persons with type 2 diabetes: results from the DIAB-CORE Consortium including six German population-based studies

**DOI:** 10.1186/1475-2840-11-50

**Published:** 2012-05-08

**Authors:** Ina-Maria Rückert, Michaela Schunk, Rolf Holle, Sabine Schipf, Henry Völzke, Alexander Kluttig, Karin-Halina Greiser, Klaus Berger, Grit Müller, Ute Ellert, Hannelore Neuhauser, Wolfgang Rathmann, Teresa Tamayo, Susanne Moebus, Silke Andrich, Christa Meisinger

**Affiliations:** 1 Institute of Epidemiology II, Helmholtz Zentrum München, German Research Center for Environmental Health (GmbH), Ingolstädter Landstrasse 1, D-85764, München/Neuherberg, Germany; 2 Institute of Health Economics and Health Care Management, Helmholtz Zentrum München, German Research Center for Environmental Health (GmbH), Neuherberg, Germany; 3 Institute for Community Medicine, Ernst Moritz Arndt-University, Greifswald, Germany; 4 Institute of Medical Epidemiology, Biostatistics and Informatics, Martin-Luther-University Halle-Wittenberg, Halle (Saale), Germany; 5 Division of Cancer Epidemiology, German Cancer Research Centre, Heidelberg, Germany; 6 Institute of Epidemiology and Social Medicine, University of Muenster, Muenster, Germany; 7 Department of Epidemiology and Health Reporting, Robert-Koch-Institute, Berlin, Germany; 8 Institute of Biometrics and Epidemiology, German Diabetes Center, Leibniz Center for Diabetes, Research at Heinrich-Heine-University Düsseldorf, Düsseldorf, Germany; 9 Institute of Medical Informatics, Biometry and Epidemiology, University Hospital of Essen, University of Duisburg-Essen, Essen, Germany; 10 MONICA/KORA Myocardial Infarction Registry, Central Hospital of Augsburg, Augsburg, Germany

**Keywords:** Type 2 Diabetes, Hypertension, Dyslipidemia, Adherence to guidelines, Pharmacological treatment

## Abstract

****Background**:**

Although most deaths among patients with type 2 diabetes (T2D) are attributable to cardiovascular disease, modifiable cardiovascular risk factors appear to be inadequately treated in medical practice. The aim of this study was to describe hypertension, dyslipidemia and medical treatment of these conditions in a large population-based sample.

****Methods**:**

The present analysis was based on the DIAB-CORE project, in which data from five regional population-based studies and one nationwide German study were pooled. All studies were conducted between 1997 and 2006. We assessed the frequencies of risk factors and co-morbidities, especially hypertension and dyslipidemia, in participants with and without T2D. The odds of no or insufficient treatment and the odds of pharmacotherapy were computed using multivariable logistic regression models. Types of medication regimens were described.

****Results**:**

The pooled data set comprised individual data of 15, 071 participants aged 45–74 years, including 1287 (8.5%) participants with T2D. Subjects with T2D were significantly more likely to have untreated or insufficiently treated hypertension, i.e. blood pressure of > = 140/90 mmHg (OR = 1.43, 95% CI 1.26-1.61) and dyslipidemia i.e. a total cholesterol/HDL-cholesterol ratio > = 5 (OR = 1.80, 95% CI 1.59-2.04) than participants without T2D. Untreated or insufficiently treated blood pressure was observed in 48.9% of participants without T2D and in 63.6% of participants with T2D. In this latter group, 28.0% did not receive anti-hypertensive medication and 72.0% were insufficiently treated. In non-T2D participants, 28.8% had untreated or insufficiently treated dyslipidemia. Of all participants with T2D 42.5% had currently elevated lipids, 80.3% of these were untreated and 19.7% were insufficiently treated.

****Conclusions**:**

Blood pressure and lipid management fall short especially in persons with T2D across Germany. The importance of sufficient risk factor control besides blood glucose monitoring in diabetes care needs to be emphasized in order to prevent cardiovascular sequelae and premature death.

## **Background**

Atherosclerosis accounts for most deaths in people with type 2 diabetes (T2D) and the age adjusted relative risk of coronary artery disease and peripheral arterial disease has been reported to be threefold higher than in the general population [1-3]. In a population-based study conducted by Haffner et al. 1998 [4], the 7-year incidence of first myocardial infarction or death was 18.8% in T2D patients compared to 3.5% in non-T2D persons. Cerebrovascular disease is also more common in subjects with T2D due to limited cerebrovascular arterial circulation and cerebral hemodynamic and vascular derangements [5]. As a precursor of frank T2D, insulin resistance has been shown to increase the risk of cardiovascular events in non-diabetic patients without history of myocardial infarction or stroke [6] and the risk of new cardiovascular events in non-diabetic patients with manifest arterial disease [7]. The findings indicate that insulin resistance per se and independently of other components of the metabolic syndrome, including inflammation, has an influence on cardiovascular risk.

Hypertension and dyslipidemia are often associated with insulin resistance, frequent in T2D and enhance the risk of macrovascular complications like coronary artery disease and stroke as well as microvascular sequelae like retinopathy and nephropathy [8].

Thus, subjects with T2D particularly profit from lifestyle modifications and medication therapy aiming at a normotensive blood pressure and low lipid concentrations in the blood [1].

Nevertheless, several studies in Germany and other countries have shown that patients in primary care [9-11] and particularly patients with T2D are not adequately treated with antihypertensive and lipid lowering medications. In general, about 50% of patients do not reach a blood pressure of < -140/90 mmHg and about the same proportion of patients have dyslipidemia, depending on the definition used [12-18].

### **Objectives**

While patient-related data from primary care and patients-registries are available demonstrating an under-treatment of T2D patients in clinical practice, results from population-based studies are scarce. Such data, however, are important to generalize knowledge on the treatment status of specific populations and to identify regional differences in the quality of health care. Moreover, studies using clinical practice data probably underestimate frequencies since well-treated patients are more likely to be recruited.

In the current analysis, we used pooled data from the DIAB-CORE data set to assess the frequencies of risk factors associated with cardiovascular disease (CVD), co-morbidities, medication intake, adequate and insufficient treatment in participants with and without T2D from Germany. Our pre-specified hypothesis based on current literature was that blood pressure and lipid levels are not sufficiently controlled, particularly in patients with T2D.

## **Methods**

### **Study design and setting**

The DIAB-CORE Consortium has been launched in order to establish a joint pool of population-based data on persons with and without diabetes. Six studies covering regions in Germany were combined (from north to south): the Study of Health in Pomerania (SHIP, Greifswald), the Dortmund Health Study (DHS, Dortmund), the Cardiovascular Disease, Living and Ageing (CARLA, Halle-Wittenberg) Study, the Heinz Nixdorf-Risk Factors, Evaluation of Coronary Calcification, and Lifestyle (HNR Recall, Bochum, Essen, Mülheim a. d. Ruhr) Study, the Cooperative Health Research in the Region of Augsburg (KORA, Augsburg) Study, and the nationwide German National Health Interview and Examination Survey 1998 (GNHIES98, Germany), see Table 1.

**Table 1:** Studies included in the pooled DIAB-CORE sample (45–74 years), north to south

**Study**	**Region**	**Study period**	**N (%)**	**Age (years) mean (SD)**	**Women (%)**	**T2D (%)**
**SHIP**^**a**^	North-east Germany (West Pomerania)	1997–2001	2247 (14.9)	59.0 (8.3)	1128 (50.2)	251 (11.2)
**DHS**^**b**^	West Germany (Dortmund)	2003-2004	883 (5.9)	60.1 (8.5)	447 (50.6)	87 (9.9)
**CARLA**^**c**^	East Germany (Halle)	2002–2006	1382 (9.2)	60.2 (7.9)	651 (47.1)	174 (12.6)
**NHR**^**d**^	West Germany (Bochum, Essen, Mühlheim an der Ruhr)	2000-2003	4734 (31.4)	59.6 (7.8)	2379 (50.3)	350 (7.4)
**KORA**^**e**^	South Germany (Augsburg region)	1999–2001	2442 (16.2)	58.9 (8.4)	1227 (50.3)	146 (6.0)
**GNHIES98**^**f**^	Nationwide	1997–1999	3383 (22.5)	58.0 (8.0)	1749 (51.7)	279 (8.3)
**Total**	Germany	1997–2006	15071	59.1 (8.1)	7581 (50.3)	1287 (8.5)

^a^SHIP: Study of Health in Pomerania; ^b^DHS: Dortmund Health Study; ^c^CARLA: Cardiovascular Disease, Living and Ageing in Halle; ^d^HNR: Heinz Nixdorf-Recall; ^e^KORA (Survey S4): Cooperative Health Research in the Region of Augsburg; ^f^GNHIES98: German National Health Interview and Examination Survey 1998.

All studies were conducted between 1997 and 2006 and used similar instruments, questionnaires and medical measurements to collect data. Detailed descriptions of study designs, samples and procedures are available elsewhere [19-25]. Ethical approval and written informed consent was obtained for each study.

### **Variables**

#### ***Age***

Only participants aged 45 to 74 years were included in the pooled data set, representing the common intersection of all studies.

#### ***School education***

A binary variable was created contrasting individuals with high and middle educational graduation (higher educational entrance qualification, advanced technical college entrance qualification, general certificate of secondary education or polytechnic grammar school) versus low educational graduation (no school certificate or junior high school only).

#### ***Income***

Participants were asked to choose their appropriate income class. The midpoints of these classes were used to define the variable household net income separately for each regional study. The lowest income group was defined as less than 60% of the median income in the individual study and was compared to the other groups.

#### ***Smoking***

Two categories (current vs. ex- and never smoker) were defined to differentiate risk types. A current smoker smoked at least one cigarette per day. An ex-smoker had quit smoking at least one year ago, otherwise he or she was regarded as a current smoker.

#### ***Body mass index***

Body mass index was calculated as weight in kilograms divided by height in square meters (unit kg/m^2^).

#### ***Physical activity***

In all studies physical activity was assessed by self-report only. A threshold of < = 1 h per week was determined as physical inactivity. Assessment of physical activity included all kinds of exercise training but did not comprise low level exercise such as stepping stairs or walking, as this type of exercise was not assessed in all studies.

#### ***T2D***

T2D was defined based on self-report of physician's diagnosis or self-reported intake of oral anti-diabetic agents, insulin or a combination of both. Some studies lacked information on diabetes type. Thus, in order to exclude participants who probably had Type 1 diabetes, self-reported age at diagnosis of diabetes was used, and only those patients with an age at diagnosis of > 30 years were included in the T2D group.

#### ***Hypertension***

Hypertension was defined using the mean of the second and third blood pressure measurements (the first and second measurements in DHS) conducted at the study centres with systolic blood pressure > =140 and/or diastolic blood pressure > =90 mmHg, or intake of anti-hypertensive medication in participants with physician’s diagnosis of hypertension (“awareness”). Participants with hypertension were categorized into one of the following four subgroups: (1) aware (with physician’ diagnosis) and controlled treated to target levels of < 140/90 mmHg, (2) aware and treated, but not reaching target blood pressure values of < 140/90 mmHg, i.e. insufficiently treated, (3) aware, but not treated, (4) unaware of hypertension. Thus, “awareness” of hypertension applied to participants in categories 1, 2 and 3, “treatment” applied to those in categories 1 and 2 and “control” to those in category 1[26].

Hypertension guidelines [27-31] launched after 2000, advocate treating blood pressure to < 140/90 mmHg in persons without diabetes and < 130/80 mmHg in persons with diabetes. The lower threshold for patients with diabetes or persons at high risk for cardiovascular disease, respectively, is relatively new and has been criticized by recent publications [32]. Due to intense medical treatment, subjects are more likely to experience side effects, such as hypotension, hypokalemia and worsening of renal function. German guidelines also question the benefit of a lower blood pressure goal because of inconsistent clinical evidence [33]. Thus, we chose a blood pressure of < 140/90 mmHg as a goal in both participants with and without diabetes.

#### ***Dyslipidemia***

Total cholesterol, high-density lipoprotein (HDL) cholesterol and low-density lipoprotein (LDL) cholesterol levels were measured from random blood samples. Dyslipidemia was defined analogous to hypertension using information on lipid-lowering medication intake, self-reported information on physician's diagnoses and a total cholesterol to HDL cholesterol ratio (TC/HDL) of  > = 5 [34]. According to the Adult Treatment Panel III 2001 of the US National Institutes of Health [35], diagnosis of dyslipidemia should be based on LDL cholesterol levels (> 100 mg/dl in high risk persons, > 160 mg/dl in persons without additional CVD risk factors). However, if the testing opportunity is non-fasting, only total cholesterol and HDL cholesterol levels are usable. Kinosian et al. [36] suggested that the ratio of total cholesterol to HDL cholesterol has a better potential to discriminate people at high risk of future cardiovascular disease than total cholesterol or LDL cholesterol values respectively.

#### ***Burden of CVD***

Self-reported data on myocardial infarction and stroke (“Did you ever have a myocardial infarction/stroke, diagnosed by a physician?”) was assessed identically in all studies. Information on angina pectoris (Rose questionnaire reference) and intermittent claudication was also collected, although the questions were not identical across the studies. However, we tried to achieve the best possible harmonization by using all available information. Prevalent CVD was defined as presence of self-reported MI, stroke, angina or claudication.

#### ***Anti-hypertensive medication***

All study participants were asked to bring original packaging of their medications used during the last seven days to the examination. Unique pharmaceutical identifiers, names etc. were recorded and ATC (Anatomical Therapeutic Chemical Classification System) codes were assigned accordingly. The variable “anti-hypertensive medication” included any prescription of medication belonging to the ATC subgroups C02 (antihypertensives), C03 (diuretics), C04 (peripheral vasodilators), C07 (beta blocking agents), C08 (calcium channel blockers) and C09 (agents reacting on the renin-angiotensin system).

#### ***Lipid-lowering medication***

Medications of the ATC subgroup C10 (lipid modifying agents) were included in the variable “lipid-lowering medication”.

### **Participants**

Our pooled data set included 1287 participants with T2D and 13784 participants without T2D aged 45 to 74 years from major regions in Northeast, Middle, West- and South-Germany. Two-stage cluster sampling or stratified random sampling were used. In the nationwide survey GNHIES98, 3% non-German but German speaking citizens were included. KORA, SHIP, CARLA and HNR focused on participants of German nationality, in DHS nationality was not used as an inclusion criterion. Overall response ranged between 56% and 69%.

Since physician's diagnosis of dyslipidemia had not been assessed in DHS, the study was not included in analyses using the combined variable of medication intake, laboratory measurements and physician's diagnosis to classify participants with dyslipidemia. Because of missing values, participants had to be excluded from the hypertension sub-analyses (n = 358), and from the dyslipidemia sub-analyses (n = 1460) using these combined variables. Participants were excluded from the logistic regressions on current hypertensive measurements (n = 26) and current dyslipidemia (n = 337), due to missing information on blood pressure or lipid measurements. Participants (n = 4612) with complete variable information on the combined hypertension variable, who had a positive physician’s diagnosis and used anti-hypertensive medication were included to assess the frequencies of medication classes.

### **Statistical analyses**

Men and women with diabetes and participants without diabetes were compared with respect to their lifestyle factors, cardiovascular burden, clinical measurements and medications. For continuous variables means and standard deviations (SD) were calculated, while categorical variables were described as percentages. Differences between groups were tested using t-tests and Wilcoxon tests (continuous variables) or chi-square tests and univariate logistic regression models (categorical variables). Logistic regression models were calculated to identify the effects of a basic set of variables that influence odds of hypertension, dyslipidemia and medication intake, respectively.

A two-sided alpha level of 0.05 was chosen as criterion for statistical significance. All analyses were carried out using SAS, version 9.1 (SAS Institute Inc., Cary, NC, USA).

## **Results**

### **Participants**

**Figure 1:**
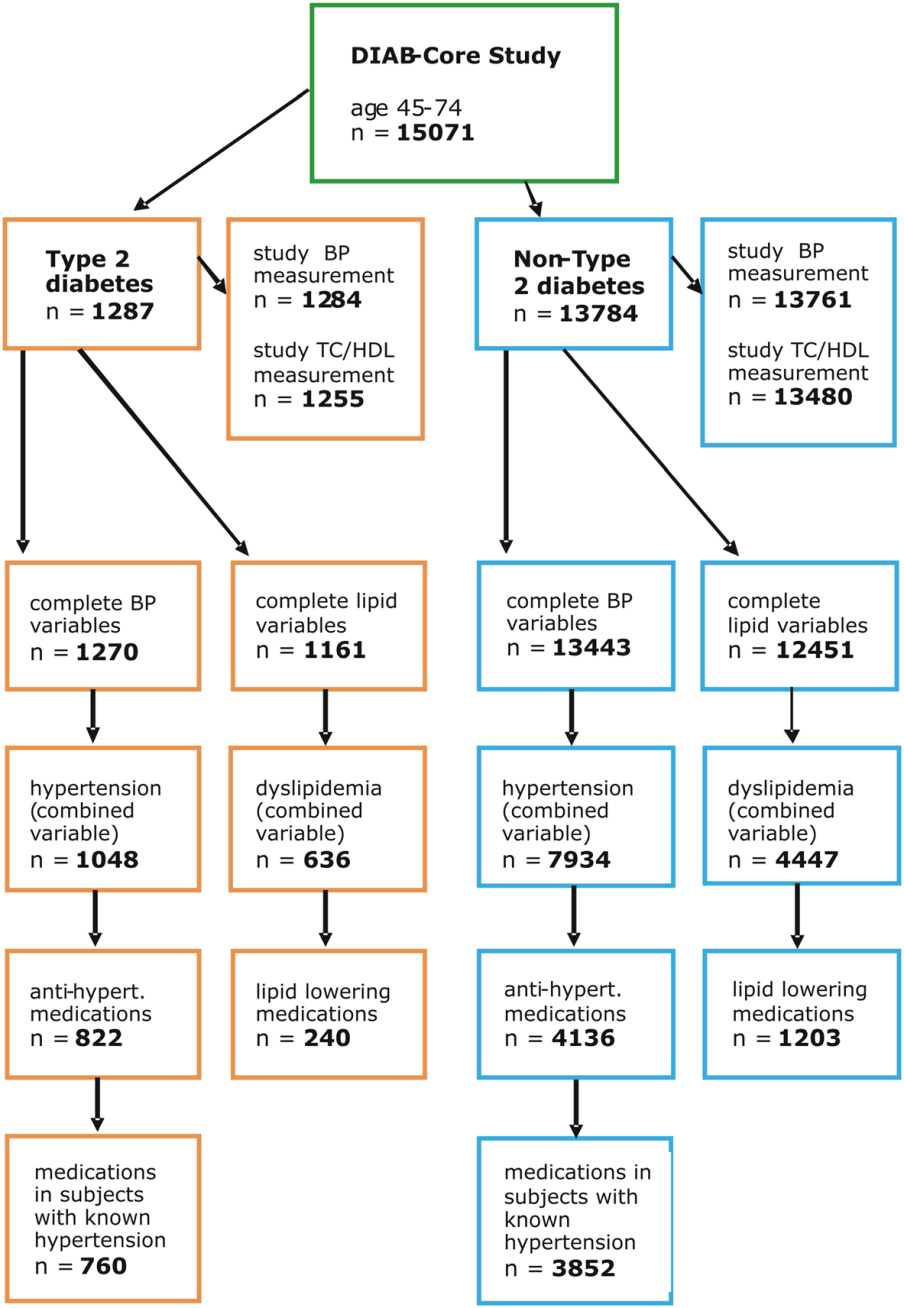
Participant selection (flow chart).

The pooled sample included 15071 participants (47.1-51.7% women across studies) aged 45–74 years (Table 1). Overall 1287 (8.5%) participants had T2D (706 (54.9%) men and 581 (45.1%) women). The proportion of participants with T2D according to the standardized definition in DIAB-CORE ranged between 6.0% in KORA to 12.6% in CARLA. The national survey study reported 8.3% respondents with T2D. The mean age of all participants was 59.1 (SD = 8.1). The flow chart (Figure 1) indicates the numbers of participants within subgroups of interest.

### **Study characteristics**

Participants with T2D were older than participants without T2D (Table 2). Significantly more men than women presented with T2D. On average, participants with T2D had a BMI of 30.8 kg/m^2^, compared to 27.9 kg/m^2^ in participants without diabetes. Participants with T2D smoked less. However, they were also less physically active, were characterized by lower income and lower educational status, particularly women. Subjects with T2D had higher systolic and diastolic blood pressure than non-T2D participants, 82.5% were classified as hypertensive compared to 59.0% of participants without T2D. They had more favourable total cholesterol- and LDL cholesterol values but less favourable HDL cholesterol values, and were more frequently diagnosed with dyslipidemia (54.8% vs. 35.7%).

**Table 2:** Study characteristics in DIAB-CORE, age range 45-74

	**Type 2 diabetes**			**Non-type 2 diabetes**
	**Men, n = 706**	**Women, n = 581**	**All, n = 1287**	**All, n = 13784**
Age (years)	62.5 (7.2)	63.8 (7.1)^*^	63.1 (7.2)	58.7 (8.1)^◯^
Women (%)	-	-	45.1	50.8^◯^
BMI (kg/m2)	30.1 (4.8)	31.6 (5.7)^*^	30.8 (5.3)	27.9 (4.5)^◯^
BMI > = 30 (%)	41.8	58.1^*^	49.2	27.6^◯^
Smoking (%)	20.8	8.9^*^	15.5	21.3^◯^
Low physical activity (%)	73.2	71.6	72.5	58.0^◯^
Low income (%)	13.7	21.9^*^	17.3	12.5^◯^
Low education (%)	73.5	83.6^*^	78.1	63.6^◯^
Diabetes duration (years)	8.8 (7.7)	8.2 (7.2)	8.5 (7.5)	-
**Diabetes treatment (%)**				
Diet only or no treatment (%)	22.1	23.4	22.7	-
OAD only (%)	52.8	47.0	50.2	-
Insulin only (%)	15.5	18.8	17.0	-
OAD and Insulin (%)	9.7	10.8	10.2	-
**Blood pressure (mmHg)**				
Systolic BP (mm Hg)	149.1 (22.1)	145.8 (21.8)^*^	147.6 (22.0)	138.5 (21.5)^◯^
Diastolic BP (mm Hg)	85.0 (11.4)	82.1 (10.9)^*^	83.7 (11.3)	84.5 (11.4)^◯^
^⋄^**Hypertension (%)**	80.7	84.8	82.5	59.0^◯^
**current BP > = 140/90 (%)**	67.0	59.6^*^	63.6	48.9^◯^
**Cholesterol (mg/dl)**				
TC (mg/dl)	216.4 (48.0)	232.2 (48.8)^*^	223.5 (49.0)	236.2 (43.7)^◯^
LDL (mg/dl)	132.1 (37.8)	142.1 (41.5)^*^	136.6 (39.8)	149.5 (39.5)^◯^
HDL (mg/dl)	46.3 (14.0)	54.0 (16.6)^*^	49.8 (15.7)	58.4 (17.7)^◯^
^⋄⋄^**Dyslipidemia (%)**	56.7	52.5	54.8	35.7^◯^
**current TC/HDL ratio > = 5 (%)**	45.7	36.5^*^	41.6	28.8^◯^
Myocardial infarction (%)	13.7	7.7^*^	11.0	4.1^◯^
Stroke (%)	8.4	5.5^*^	7.1	2.4^◯^
Claudicatio intermittens (%)	13.3	10.6	12.1	3.8^◯^
Angina pectoris (%)	16.4	20.6^*^	18.3	9.3^◯^
**History of CVD (%)**	34.3	32.3^*^	33.4	15.5^◯^

Numbers are means (SD) or percentages and relate to the number of subjects available for analysis. BMI: body mass index, OAD: oral anti-diabetic medication, TC: total cholesterol, LDL: low-density lipoprotein, HDL: high-density lipoprotein, CVD: cardiovascular disease, BP: blood pressure.

^⋄^BP > = 140/90 mmHg or using anti-hypertensive medication.

^⋄⋄^TC/HDL > = 5 or using lipid-lowering medication.

^*^Test of the difference between T2D men and T2D women, p <0.05.

^◯^Test of the difference between all T2D subjects and all non-T2D subjects, p < 0.05.

Manifest cardiovascular diseases were generally about twice to threefold as common among study participants with T2D with 11.0% who had previously experienced myocardial infarction compared to 4.1% in participants without T2D (Table 2). 7.1% of subjects with T2D reported a stroke versus 2.4% of participants without T2D. Similar differences were observed for intermittent claudication and angina pectoris.

### **Frequency and medical treatment of hypertension**

**Figure 2:**
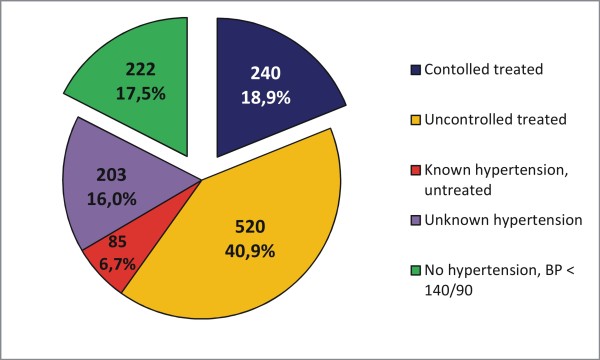
** Frequencies of controlled treated, uncontrolled, known but untreated and unknown hypertension in participants with T2D.** Hypertension was defined as BP > = 140/90 mmHg, N = 1270.

Of participants with T2D who had complete variable information on medication intake and study measurements (N = 1270), 82.5% had hypertension compared to 59.0% in non-T2D participants. Considering the four subgroups, 18.9% of all participants with T2D were controlled treated (1) with mean blood pressure: 125/77 mmHg, 40.9% were insufficiently treated (2) with mean blood pressure: 159/91 mmHg, 6.7% were aware of the disease but not treated (3) with mean blood pressure: 159/95 mmHg and 16.0% were not aware (4) with mean blood pressure: 151/91 mmHg. Anti-hypertensive medications were taken by 69.7% of subjects, by 59.8% of subjects with known hypertension, i.e. 9.9% probably used these medications for a different indication. On site measurements of  > =140/90 mmHg, e.g. untreated or insufficiently treated hypertension was recorded in 63.6%. In comparison, the percentage of participants without T2D with untreated or insufficiently treated hypertension was 48.9% of 13443 subjects with complete variable information (Table 3, Figure 2). Anti-hypertensive medications were used by 34.5% of non-T2D participants and by 28.7% with diagnosed hypertension.

**Table 3:** Frequencies of hypertension, dyslipidemia, and adequacy of treatment in participants with and without T2D

	**Type 2 diabetes**			**Non-type 2 diabetes**
	**Men, n = 706**	**Women, n = 581**	**All, n = 1287**	**All, n = 13785**
**Complete cases hypertension**	**n(cc) = 698**	**n(cc) = 572**	**n(cc) = 1270**	**n(cc) = 13443**
No hypertension	135 (19.3)	87 (15.2)	222 (17.5)	5509 (41.0)^◯^
**Hypertension**	563 (80.7)	485 (84.8)	1048 (82.5)	7934 (59.0)^◯^
(1) Controlled treated, BP < 140/90	97 (13.9)	143 (25.0)*	240 (18.9)	1357 (10.1)^◯^
(2) Uncontrolled treated, > = 140/90	271 (38.8)	249 (43.5)*	520 (40.9)	2495 (18.6)^◯^
(3) Known, but not treated, > = 140/90	51 (7.3)	34 (5.9)	85 (6.7)	1178 (8.8)^◯^
(4) Unknown, > = 140/90	144 (20.6)	59 (10.3)*	203 (16.0)	2904 (21.6)^◯^
**Untreated or insufficiently treated (groups 2, 3, 4)**	**466 (66.8)**	342 (59.8)*	**808 (63.6)**	**6577 (48.9)**^◯^
GNHIES98	101 (72.1)	94 (67.6)	195 (69.9)	1904 (61.4)^◯^
CARLA	72 (75.8)	42 (53.9)*	114 (65.9)	668 (55.3)^◯^
DHS	39 (75.0)	24 (68.6)	63 (72.4)	503 (63.3)
KORA	49 (61.3)	37 (56.9)	86 (59.3)	898 (39.3)^◯^
HNR	107 (52.7)	66 (49.6)	173 (51.5)	1504 (36.8)^◯^
SHIP	98 (76.6)	80 (65.0)*	178 (70.9)	1110 (55.7)^◯^
**Complete cases dyslipidemia**	**n(cc) = 639**	**n(cc) = 522**	**n(cc) = 1161**	**n(cc) =12451**
No dyslipidemia	277 (43.4)	248 (47.5)	525 (45.2)	8004 (64.3)^◯^
**Dyslipidemia**	362 (56.7)	274 (52.5)	636 (54.8)	4447 (35.7)^◯^
(1) Controlled treated , TC/HDL <5	65 (10.2)	78 (14.9)	143 (12.3)	861 (6.9)^◯^
(2) Uncontrolled treated, > = 5	43 (6.7)	41 (7.9)	84 (7.2)	299 (2.4)^◯^
(3) Known, but not treated, > = 5	114 (17.8)	85 (16.3)	199 (17.1)	1535 (12.3)^◯^
(4) Unknown, > = 5	140 (21.9)	70 (13.4)*	210 (18.1)	1752 (14.1)^◯^
**Untreated or insufficiently treated (groups 2, 3, 4)**	**297 (46.5)**	196 (37.6)*	**493 (42.5)**	**3586 (28.8)**^◯^
GNHIES98	73 (54.9)	62 (48.8)	135 (51.9)	1040 (35.3)^◯^
CARLA	34 (35.8)	18 (23.1)	52 (30.1)	280 (23.3)
(DHS)^** ◖**^	(15 (31.3))	(7 (22.6))	(22 (27.9)	(105 (14.7)
KORA	39 (48.8)	24 (38.1)	63 (44.1)	675 (29.7)^◯^
HNR	87 (42.9)	41 (31.1)*	128 (38.2)	996 (24.5)^◯^
SHIP	64 (50.0)	51 (41.5)	115 (45.8)	600 (30.4)^◯^

BP: blood pressure, TC: total cholesterol, HDL: high density lipoprotein n(cc) = number of complete cases concerning variables used for these statistics, i.e. physician's diagnosis, medication intake and BP measurement or lipid measurement, respectively.

*Test of the difference between T2D men and T2D women, p <0.05.

^◯^Test of the difference between all T2D subjects and all non-T2D subjects, p < 0.05.

^◖^without consideration of physician's diagnosis because this variable was missing in DHS.

### **Frequency and medical treatment of dyslipidemia**

**Figure 3:**
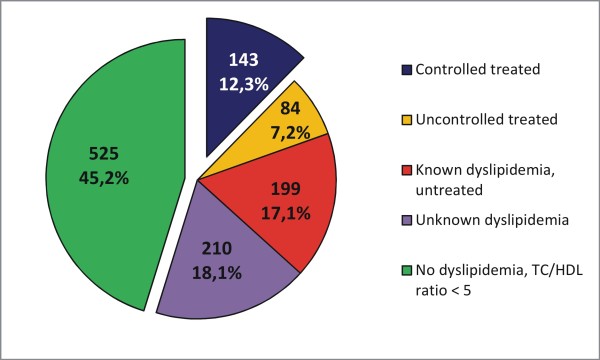
** Frequencies of controlled treated, uncontrolled, known but untreated and unknown dyslipidemia in participants with T2D.** Legend: Dyslipidemia was defined as TC/HDL Ratio > = 5, N = 1161.

Of all T2D participants with complete data on intake of lipid modifying medication, physician's diagnosis and laboratory measurements (n = 1161, without DHS), 54.8% were classified as having dyslipidaemia. The four subgroups displayed the following frequencies: 12.3% were controlled treated (1) with mean TC/HDL: 3.7, 7.2% were insufficiently treated (2) with mean TC/HDL: 6.3, 17.1% were aware of the disease but not treated (3) with mean TC/HDL: 6.4 and 18.1% were not aware (4) with mean TC/HDL: 6.1. Currently elevated lipids (TC/HDL > =5), i.e. untreated or insufficiently treated dyslipidemia was recorded in 42.5%. Lipid lowering medications were taken by 23.2% of participants (and by 19.6% with known dyslipidemia, i.e. 3.6% probably used these medications for a different indication). In non-T2D study participants, 35.7% of 12450 with complete cholesterol, physician and medication variables had dyslipidemia, 28.8% were untreated or insufficiently treated. Lipid lowering medications were used by 10.7% of non-T2D participants (by 9.3x% with diagnosed dyslipidemia) (Table 3, Figure 3).

### **Basic factors that influence treatment goals and the odds of using medication**

Fitting logistic regression models including all study participants without missing values (N = 15045), we found that participants with diabetes had significantly higher odds of failing to reach the blood pressure target (OR = 1.43, 95% CI 1.26-1.61) (Table 4). Age and male sex were also significantly associated with unfavourable blood pressure levels. Using the nationwide and oldest study, GNHIES98, as reference, all regional studies except DHS achieved more favourable results. The interaction term of age and sex was significant, indicating that in women the odds of hypertension increased more steeply with increasing age than in men.

**Table 4:** Logistic Regression Model: Untreated or insufficiently treated hypertension or dyslipidemia, respectively, in participants with and without T2D (only participants with missing study measurements were excluded)

		**Blood pressure > = 140/90 mmHg n = 15045, n (BP > = 140/90 mmHg) = 7551**			**Total cholesterol/HDL > =5 n = 14735, n (TC/HDL > = 5) = 4300**		
**Effect**		**OR**	**95% CI**	**p-value**	**OR**	**95% CI**	**p-value**
Diabetes	yes	1.43	1.26-1.61	<0.0001	1.80	1.59-2.04	<0.0001
Age	year	1.05	1.04-1.05	<0.0001	1.01	1.00-1.01	0.0444
Sex	male	1.79	1.67-1.91	<0.0001	3.01	2.79-3.25	<0.0001
Study							
	KORA	0.38	0.34-0.42	<0.0001	0.74	0.66-0.84	<0.0001
	CARLA	0.68	0.60-0.78	<0.0001	0.48	0.42-0.56	<0.0001
	DHS	0.99	0.85-1.17	0.9446	0.29	0.24-0.36	<0.0001
	HNR	0.34	0.31-0.37	<0.0001	0.56	0.51-0.62	<0.0001
	SHIP	0.76	0.68-0.85	<0.0001	0.77	0.68-0.87	<0.0001
	GNHIES98	Ref.			Ref.		
**Interactions**							
Diabetes*Sex				0.2494			<.0001
Age*Sex				0.0013			<.0001
Age*Study				0.0032			0.0042
Sex*Study				0.0002			0.0133
**Stratification**							
Diabetes (yes)	Men				1.37	1.16-1.61	0.0002
	Women				2.32	1.91-2.82	<.0001
Age (year)	Men	1.04	1.03-1.04	<.0001	0.99	0.99-1.00	0.0062
	Women	1.05	1.05-1.06	<.0001	1.03	1.02-1.04	<.0001

Stratified analyses were also adjusted for age and study or diabetes and study respectively.

Dyslipidemia (n = 14735) was also positively associated with diabetes status (OR = 1.80, 95% CI 1.59-2.04) and male sex (OR = 3.01, 95% CI 2.79-3.25). All regional studies reported a significantly lower number of participants with TC/HDL > = 5 than GNHIES98. Interaction of diabetes and sex showed that women with diabetes had a much higher odds of dyslipidemia (OR = 2.32, 95% CI 1.91-2.82) than men with diabetes (OR = 1.37, 95% CI 1.16-1-61). Moreover, in women the odds increased with increasing age, while in men, the odds decreased slightly.

The odds of anti-hypertensive treatment in hypertensive patients (n = 8982) were clearly higher in subjects with T2D (OR = 2.86, 95% CI 2.44-3.35), older and female participants (Table 5). Compared to GNHIES98, participants of CARLA, HNR and SHIP used anti-hypertensive medication more frequently. Men received medication more likely with increasing age than women.

**Table 5:** Logistic Regression Model: Medical treatment in participants with hypertension (including unknown hypertension) or dyslipidemia (including unknown dyslipidemia), respectively

		**Anti-hypertensive medication n = 8982, n (med) = 4958**			**Lipid-lowering medication n = 5083, n (med) = 1443**		
**Effect**		**OR**	**95% CI**	**p-value**	**OR**	**95% CI**	**p-value**
Diabetes	yes	2.86	2.44-3.35	<0.0001	1.38	1.15-1.65	0.0006
Age	year	1.07	1.06-1.07	<0.0001	1.06	1.05-1.07	<0.0001
Sex	male	0.64	0.58-0.70	<0.0001	0.59	0.52-0.67	<0.0001
Study							
	KORA	1.33	1.15-1.54	<0.0001	1.25	1.01-1.53	0.0383
	CARLA	1.95	1.66-2.30	<0.0001	1.94	1.53-2.50	<0.0001
	DHS	0.93	0.77-1.12	0.4219	*-	-	-
	HNR	1.96	1.73-2.22	<0.0001	1.96	1.64-2.33	<0.0001
	SHIP	1.68	1.47-1.93	<0.0001	1.27	1.03-1.56	0.0232
	GNHIES98	Ref.			Ref.		
**Interactions**							
**Effect**		**OR**	**95% CI**	**p-value**	**OR**	**95% CI**	**p-value**
Age*Diabetes				0.0581			0.0037
Age*Sex				0.0001			0.6613
Age*Study				0.0006			0.0295
**Stratification**							
Age (year)	T2D				1.02	1.00-1.05	0.0593
	Non-T2D				1.06	1.05-1.07	<.0001
Age (year)	Men	1.08	1.07-1.09	<.0001			
	Women	1.05	1.04-1.06	<.0001			

*no information on physician’s diagnosis in DHS.

Stratified analyses were also adjusted for sex and study or diabetes and study respectively.

In persons with dyslipidemia (n = 5083), lipid-lowering preparations were also used more often by participants with T2D (OR = 1.38, 95%CI 1.15-1.65), older participants (OR = 1.06, 95% CI 1.05-1.07) and women (OR for men: 0.59, 95%CI 0.52-0.67). CARLA, HNR and SHIP had higher rates of lipid-lowering medication than GNHIES98. DHS was not included in this model. The odds of taking lipid-lowering medications increased significantly with increasing age in participants without T2D, but not in participants with T2D.

Some interaction terms involving the ‘individual study’ variable were also statistically significant (Tables 4 and 5).

### **Anti-hypertensive treatment regimens**

Anti-hypertensive treatment regimens were assessed in participants who were aware of their condition (subgroups 1 and 2, N = 4612) only, assuming that individuals who received medications without having a physician’s diagnosis of hypertension used these preparations for different indications (e.g. cardiac insufficiency). Participants with T2D received preparations of the ATC groups: C03 (diuretics), C08 (calcium channel blockers) and C09 (preparations acting on the renin-angiotensin system, including ACE inhibitors and angiotensin II receptor blockers), more frequently than non-T2D hypertensive participants (Table 6). Thirty-nine point nine percent of all 760 treated subjects with T2D and 56.2% of 3852 treated participants without T2D received only one preparation or preparations of one blood pressure lowering ATC group. A combination of three or more ATC groups was used by 24.5% of T2D subjects and 12.5% of non-T2D subjects.

**Table 6:** Medication groups used for treating hypertensive T2D and non-T2D participants respectively

**ATC group**		**Use in treated participants with T2D**		**Use in treated participants without T2D**		**Comment**
		**N = 760 (%) single or in combination with other preparations**	**as spreparation/ATC group**	**N = 3852 (%) single or in combination with other preparations**	**as single preparation/ATC group**	
**C02**	Anti-hypertensives	9.47	2.11	8.00	2.78	
**C03**	Diuretics	29.47	2.50	17.29*	2.52	not ideal as first line treatment since they can worsen glucose and lipid levels
**C04**	Peripheral vasodilators	4.21	0.13	2.65*	0.31	
**C07**	Beta blocking agents	44.74	8.82	52.23*	23.81*	best used as second- or third-line treatments in T2D, more efficacious in younger patients (55–60 years), not for asthma patients, because of bronchoconstrictive effects, promote weight gain, may mask hypoglycaemia
**C08**	Calcium channel blockers	33.68	5.79	27.13*	7.84*	not recommended as first-line and single treatment
**C09**	Agents acting on the renin-angiotensin system	68.95	20.53	51.43*	18.98	recommended as first-line treatment, reduce the risk for renal end points

* p for the difference between participants with T2D and participants without T2D <0.05.

Anti-hypertensive medication in participants who were classified as non-hypertensive or unknown was not considered in this analysis, assuming that they used these agents for different indications.

### **Time trends in uncontrolled hypertension**

Logistic models including the year of examination of each study participant adjusted for age, sex, diabetes and study revealed that there was some improvement of hypertension treatment during the DIAB-CORE study period, i.e. from 1997 to 2006. Thus, the odds of having blood pressure values  > =140/90 mmHg decreased per year (OR = 0.95 (95% CI 0.90-0.99, p = 0.0248). GNHIES98, as the oldest study, had the greatest percentage of participants with hypertension.

## **Discussion**

### **Key results**

Our study confirms that there is a clear gap between measured blood pressure and blood lipid recommendations and the actual management in 45 to 74 years old T2D and non- T2D subjects in the general German population. More than 60% of participants with T2D did not reach the conservative blood pressure target of 140/90 mmHg and about 40% had prevalent dyslipidemia (total cholesterol/HDL-ratio of  > =5). The analysis showed that about 70% of all T2D participants received anti-hypertensive medication, but 40% were insufficiently controlled. About 20% of the participants with T2D used lipid-lowering medication but 7% did not reach a total cholesterol/HDL-ratio of <5 despite medication intake. Approximately 80% of T2D subjects with currently elevated lipid levels were untreated. T2D patients had a worse cardiovascular profile compared to non-T2D subjects.

The choice of anti-hypertensive drugs differed between participants with and without diabetes, reflecting current recommendations [37]. Many study participants used only one ATC group.

The odds of having blood pressure values  > =140/90 mmHg decreased significantly over time. However, since the single studies differed in the frequency of hypertension and the individual study periods overlapped only in part, the effect was difficult to separate from the study effect and will be studied in more detail using longitudinal data.

We did not especially stress the differences between the individual studies, since they do not necessarily reflect regional differences in health care. The studies were not conducted simultaneously and data assessment and laboratory measurements were not standardized from the outset. The nationwide GNHIES98, SHIP and DHS had more unfavourable outcomes than the other studies which might be due to the fact that GNHIES98 and SHIP were the oldest studies and DHS had the smallest number of participants and only two blood pressure measurements.

### **Strengths and limitations**

The essential strength of our study is the large population-based sample drawn from the general German population aged 45 to 74 years and the fact that both, laboratory measurements and information on medication intake were available.

Due to the pooling process only similarly collected and coded data of all six studies could be used and the least common denominator had to be found. Therefore, the definition of diabetes was based on self-report of physician’s diagnosis and treatment with anti-diabetic agents rather than on clinical diagnosis and medical records. Blood pressure was calculated using the mean of the second and third measurements in all studies except for DHS, where only two measurements were performed and used to calculate the mean. This might distort the frequency in DHS and contribute to the high proportion of participants with 64.2% having a blood pressure  > = 140/90 mmHg compared to 49.3% in the other studies.

Moreover, measurements of blood pressure and lipids based on a single testing opportunity present evidence for the respective condition, but are not equal to a clinical diagnosis with repeated measurements. We cannot exclude cases of ‘white coat hypertension’, i.e. elevated blood pressure owing to the excitement of the unfamiliar situation.

Finally, all study participants were asked to bring packages of their medications to the study centres. However, due to non-compliance and forgetfulness it is possible that fewer packages were documented than had actually been prescribed. We might thus have underestimated medication intake, consequently overestimated the number of participants without treatment and probably underestimated the number of participants with insufficient treatment.

### **Generalization**

The results of our population-based study fortify the findings of patient-based German and international studies. Recently, Berthold et al. [12] described that approximately 60% of T2D patients from the German T2DSD-registry DUTY had uncontrolled systolic blood pressure  > = 140 mmHg and about 50% had uncontrolled LDL cholesterol values > = 3.4 mmol/l. These proportions differed slightly with atherosclerotic disease location. The German ESTHER Study published in 2008 found that 78% of diabetes patients had hypertension diagnosed by a physician and only 12.8% of those who received anti-hypertensive pharmacotherapy achieved blood pressure levels below 130/85 mmHg. Physician diagnosed dyslipidemia was reported in 50% of all patients [17].

A nationwide French survey conducted in 2001 and involving 410 diabetologists found that the target blood pressure of < 140/80 mmHg was attained by 29% of patients and 58% had LDL values of < than 1.3 g/l. Control of blood pressure and LDL was not considered to be optimal [38].

Similarly, the authors of a Canadian study [13] concluded that T2D patients with cardiovascular co-morbidities are insufficiently treated with medication, perhaps because of the “glucocentric view” of diabetes. They focused on antiplatelet agents, statins and ACE inhibitors. Godley et al. [14] used insurance claims data of 977 hypertensive T2D patients in the US. Only 19.7% reached the stricter blood pressure goal of < 130/85 mmHg and 52% had dyslipidemia. A recently published US investigation by DeGuzman et al. [39] including 926 high risk patients with diabetes and concomitant atherosclerotic CVD found that although the vast majority of patients were prescribed recommended drug therapy and mean cholesterol and BP values were satisfactory, the percentage of patients actually treated to goals of current guidelines was moderate. About 40% had LDL values < = 70 mg/dl and about 60% reached a systolic BP of  < = 130 mmHg.

Finally, data from 9,167 participants of the US NHANES (National Health and Nutrition Examination Survey) survey [40] showed that alongside an increasing prevalence of diabetes from 1999 to 2008 the frequency of self reported use of lipid lowering medication increased significantly. Accordingly, the proportion of participants reaching the LDL cholesterol goal of < 100 mg/dl also increased significantly from about 30% to about 50%. Although the use of antihypertensive preparations increased significantly from about 35% to about 60%, there was no change in the proportion of participants achieving the BP goal of < = 130/80 mmHg (about 50%). Moreover, only one in four people with diabetes attained both the LDL and BP targets simultaneously.

The scientific community engaged in health care management strongly postulates an aggressive treatment of dyslipidemia and hypertension and advocates the widespread use of drugs to effectively improve mortality and morbidity rates in patients at risk [41]. A large number of blood pressure lowering preparations is available today. The choice of agents depends on individual intolerances and the therapeutic effect that varies among subjects. However, though general recommendations for T2D patients exist, there is large diversity in diabetes care programs [42] and uncertainty as to which medication classes are most suitable for patients with diabetes. Usually more than one preparation is needed to achieve the target value [1].

In general, ACE inhibitors should be used first, accompanied by diuretics depending on the presence of co-morbidities. Yet diuretics alone are suspected to negatively influence blood glucose [43]. Beta blockers, AT1 blockers and calcium antagonists are recommended as well [2,44]. Beta blockers are believed to mask hypoglycaemia in patients with T2D, though evidence suggests that that is not the case [45].

Further reductions of blood pressure target values seem not to be advisable though [32]. Recently, the ACCORD study has given evidence that the reduction of systolic blood pressure to 120 mmHg did not reduce the primary endpoint (a composite of stroke, myocardial infarction and cardiovascular death) compared with the control group, in which a systolic blood pressure of 140 mmHg was targeted [46]. Due to severe adverse reactions caused by anti-hypertensive medication the overall mortality rate was even higher. Moreover, some renal markers were alarmingly impaired. The number of stroke cases, though, could be lowered by 41%. The results of the ACCORD lipid substudy were similarly disappointing [47]: Tight control of triglycerides and HDL cholesterol values was achieved with a combination of fenofibrates and statins. The endpoints were not significantly reduced.

Statins are usually prescribed to treat dyslipidemia. They are regarded as safe, provide significant cardiovascular benefits in different populations including the elderly and patients with diabetes, and may halt or slow atherosclerotic disease progression [48]. Recently, concerns have been raised that statins may increase the risk of developing diabetes in postmenopausal women [49] and with intensive-dose treatment compared to moderate-dose treatment [50]. However, the authors concluded that the mechanisms remain unclear and the putative risk needs to be balanced to well-known benefits.

Over and above, insufficient blood pressure and lipid control are not exclusively due to insufficient prescription of medication but to various factors related to the patient and the physician. Important aspects are insufficient awareness and motivation of the patient, reluctance to initiate lifestyle changes, poor compliance (e.g. because of forgetfulness, tolerability problems due to adverse side effects, polypharmacy and dosing schedule, co-payments) and failure to modify therapy, when it is indicated such as use of combination therapy if monotherapy proves to be inadequate [51,52].

Thus, apart from medication and its design, to improve secondary prevention of cardiovascular disease in primary physician health care and especially in T2D patients, the following intervention programmes should be emphasized [14]: education sessions for practitioners, medical management guidelines, physician profiling of prescribing patterns, and blood pressure monitoring kits for patients and patient education. A prominent example are T2D disease management programs (DMPs) implemented by the German social health insurance companies in 2003 which have already shown the improvement of healthcare processes and blood pressure control [53].

According to a small study by Asimakopoulou et al. [54], T2D patients are aware of their increased cardiovascular risk and even tend to overestimate it. However, in contrast to realistic informed concern that may motivate to choose a healthier lifestyle, immoderate anxiety and fear may lead to ignorance and repression including poor compliance with medical treatment.

Therefore, individual counselling and risk communication between a health professional and the patient is essential.

## **Conclusions**

In conclusion, our analysis based on a large dataset from six population-based studies provides evidence that, among comprehensive lifestyle interventions, health care in T2D patients in Germany could be remarkably improved by focusing on cardiovascular risk factors, especially blood pressure and lipid concentration. Though numerous guidelines on the topic have been published, the transfer of theoretical knowledge to practical application appears to be very difficult. Physicians may stick to the “glucocentric view” of diabetes therapy and fail to recognise the severity of cardiovascular risk factors and co-morbidities. This approach may limit microvascular disease, but lacks the important focus on macrovascular complications.

## **Abbreviations**

ATC, Anatomical Therapeutic Chemical Classification System; BMI, body mass index; BP, blood pressure; CARLA, Cardiovascular Disease Living and Ageing Study; CVD, cardiovascular disease; DHS, Dortmund Health Study; DMP, disease management program; GNHIES98, German National Health Interview and Examination Survey; HDL, high-density lipoprotein; KORA, Cooperative Health Research in the Region of Augsburg Study; LDL, low-density lipoprotein; OAD, oral anti-diabetic medication; HNR:, Heinz Nixdorf-Risk Factors Evaluation of Coronary Calcification, and Lifestyle Study; SD, standard deviation; SHIP, Study of Health in Pomerania; T2D, type 2 diabetes; TC, total cholesterol.

## **Competing interests**

The authors declare that they have no competing interests.

## **Author’s contributions**

I-M.R. performed the statistical analyses and wrote the manuscript. C.M. and M.S. worked on pooling of the data, contributed to the discussion and reviewed/edited the manuscript. R.H. contributed to the discussion and reviewed/edited the manuscript. T.T. and W.R. worked on pooling of the data and reviewed/edited the manuscript. S.S., H.V., A.K., K.H.G., K.B., G.M., U.E., H.N, S.M. and S.A. contributed data and reviewed/edited the manuscript. All authors read and approved the final manuscript.
